# Experimental study on electrical dispersion in 3D printed resin cores reveals pore and fluid property mechanisms

**DOI:** 10.1038/s41598-025-32345-8

**Published:** 2025-12-30

**Authors:** Hongwei Shi, Shizhen Ke, Yuhang Zhang, Hao Hu, Hu Luo

**Affiliations:** College of Geophysics, China University of Petroleum, Beijing, China

**Keywords:** 3D printed cores, Rock physics experiment, Electric frequency dispersion, Pore structure, Fluid properties, Interface polarization, Geophysics, Geomagnetism

## Abstract

Quantitative analysis of the relationship between rock physical parameters (pore structure, fluid properties) and electric dispersion response is crucial for well-log interpretation and reservoir evaluation. However, the strong heterogeneity inherent in natural rocks, resulting from complex diagenetic processes, poses significant challenges in establishing suitable pore structure parameters for assessment. This study employed light-curing 3D printing technology to fabricate core samples with explicitly defined pore structures and a resolution of 49.8 μm. Initially, the complex resistivity dispersion properties of these 3D-printed cores were compared with those of heterogeneous sandstone and coal samples. While exhibiting similar dispersion trends, the resistivity magnitude and polarization frequency demonstrated marked variations attributable to differences in pore structure. Subsequently, experimental investigations were conducted over a frequency range of 40 Hz to 110 MHz to quantitatively analyze the influence of porosity, saturation, salinity, and pore-throat structure on the complex resistivity dispersion of the 3D-printed cores. Resistivity and polarization frequency exhibit a monotonic relationship with porosity, whereas they conform to a power-law relationship with respect to saturation, salinity, and pore-throat structure. Notably, variations in pore-throat diameter and length exert a significant influence on the complex resistivity dispersion properties, underscoring the critical importance of pore structure. As a pioneering effort in the literature, this study demonstrates that 3D printing technology represents a novel, feasible, and alternative laboratory testing method within the domain of electrical rock physics. Meanwhile the quantitative description of rock physical parameters and electric dispersion response can provide new ideas for geological exploration and energy development.

## Introduction

As offshore oil and gas exploration and development advance, electrical exploration methods face challenges such as the impact of reservoir heterogeneity and the accuracy of pore fluid identification. Consequently, research into the physical properties of rocks has gained increasing importance^1–3^. The electrical response of rocks provides information on reservoir saturation, lithology, and fluid type^4,5^. Crucially, the electrical dispersion characteristics of rocks are closely linked to their microstructure and fluid distribution. Numerous rock physics experiments have established regular relationships between electrical dispersion and key macroscopic parameters including porosity, permeability, pore fluid salinity, and saturation^6–9^. However, existing data primarily derive from heterogeneous natural or synthetic cores, where the inherent complexity of the pore structure inevitably influences the relationship between these physical properties and electrical dispersion behavior^10,11^. This often limits analysis to qualitative assessments. Furthermore, prevailing models for rock electrical dispersion are largely based on idealized pore geometries, the influence mechanisms of actual complex pore structures on dispersion characteristics remain unclear^12–14^. Therefore, establishing a method to explicitly characterize rock pore structure is essential to enable quantitative analysis linking physical parameters to electrical dispersion^15^. This will provide significant guidance for mechanistic studies of rock electrical dispersion.

Common methods for characterizing core pore structure include computed tomography (CT) and three-dimensional (3D) printing. CT utilizes X-ray irradiation of physical core samples to acquire data, enabling the reconstruction of three-dimensional digital rock models^16,17^. This technique provides a direct and accurate representation of the true pore structure within the core; however, it is characterized by high costs and significant time requirements^18–20^. 3D printing, also known as additive manufacturing (AM), is a process that fabricates three-dimensional objects by successively depositing printing material layer by layer. Based on digital models, this method facilitates the efficient and cost-effective production of products with complex and precise geometries and structures^21–23^. In contrast to CT scanning, 3D printing offers enhanced production flexibility and significantly reduced time and financial expenditures, while still ensuring the effective definition of pore structure information.

3D printing technology, originating in the 1980s, was first conceptualized as “rapid prototyping” by Hideo Kodama (Japan). It has since undergone significant advancements and breakthroughs in printing materials, fabrication processes, and product precision^24^, leading to widespread application across diverse fields including aerospace, medicine, construction, and education^25–27^. Within the domain of 3D printed rock cores, researchers have fabricated various samples and conducted corresponding studies. In 2016, Fereshtenejad et al. combined several printing methods based on printing options and post-processing parameters (e.g., printing orientation, layer thickness, binder saturation, heating) and analyzed their effects on the uniaxial compressive strength and stress-strain behavior of powder-based printed products^28^. While this study provided valuable guidance for research on the mechanical properties of rocks, it focused primarily on macroscopic mechanical behavior and did not address the intrinsic electrical properties of rock materials. In 2017, Song et al. integrated micro-CT technology with 3D printing, utilizing base materials such as silica sand, gypsum powder, silica beads, and resin to fabricate multiple rock core samples with resolutions up to 2 μm. They compared the porosity, permeability, tortuosity, surface roughness, and pore structure differences among these samples. Their findings indicate that gypsum offers advantages for modeling natural rock surface roughness and wettability, while resin excels at replicating pore structures^29–33^. However, the study focused primarily on parameters relevant to fluid flow (e.g., permeability) and did not extend to an in-depth investigation of the electrochemical properties governing solid–liquid interfaces. In 2019, Ardila et al. investigated the wettability preferences of sandstone analogues printed using silica sand and poly furfuryl alcohol (PFA) binder. Their results demonstrate that the strong preference of silica particles for polar fluids, combined with the affinity of the PFA binder for the oleic phase, results in the 3D printed sandstone exhibiting mixed-wet characteristics^34^. Although the wettability addressed in this study is a factor influencing the electrical properties of rocks, their discussion focused primarily on fluid distribution itself and did not extend their discussion to the associated electrical responses. In 2021, Ishutov et al. utilized reservoir rock core tomography data (1 mm diameter, 2 mm height) combined with two-photon lithography to replicate complex carbonate reservoir pores at their original scale. This approach provides unprecedented opportunities for reservoir prediction and carbon capture and storage, although a limitation is that this high-precision technique currently only replicates printed cores at millimeter scales^35^. In 2022, Sudhir Kumar et al. produced 3D printed polylactic acid (PLA) samples using laminated object manufacturing (LOM) and tested their mechanical and morphological properties, suggesting LOM’s potential utility in marine and structural engineering^36^. In 2023, Sanchez-Barra et al. evaluated the mechanical properties of 3D printed rock cores fabricated via binder jetting additive manufacturing. The results revealed significant variations in uniaxial compressive strength (ranging from 23 to 38 MPa) and Young’s modulus (ranging from 1.5 to 4.05 GPa) for the printed sandstone^37^. In 2023, Do-Hyeong Kim et al. studied changes in the mechanical strength of 3D printed composites under salt spray conditions. Their results showed a decrease in tensile strength with increasing exposure time, and the presence of shell structures reduced the rate of mechanical strength degradation, promoting the potential application of these composites in the marine industry^38^. These recent studies clearly indicate that the research focus in this field remains strongly centered on the mechanical performance and durability of materials. In summary, although 3D-printed rock cores have made significant progress in replicating the mechanical behavior and microstructure of natural rocks, the vast majority of existing studies have focused on validating them as “structural replicas” or “mechanical substitutes,” while overlooking their potential as “geophysical analogs.” In particular, whether and how the complex resistivity dispersion of rocks under alternating electric fields can be effectively replicated and quantified in 3D-printed cores remains a significant and unexplored scientific gap.

As the first attempt documented in the literature, this study applies 3D printing technology to investigate the dispersion characteristics of complex resistivity in rocks. Starting from the perspectives of porosity, throat-to-pore length ratio, and throat-to-pore diameter ratio, ten three-dimensional digital rock core models were constructed using computer-aided design software. These models were subsequently fabricated into physical samples using a high-precision stereolithography (SLA) 3D printer. Experiments were conducted to investigate the influence of parameters including porosity, saturation, salinity, and pore structure on the complex resistivity dispersion characteristics within the 3D-printed rock cores. Quantitative relationships between these factors and both resistivity and polarization frequency were analyzed. For the first time, pore structure was parameterized (using throat-to-pore diameter ratio and throat-to-pore length ratio), and the experimental regularity concerning pore structure was validated through numerical simulations using the lattice Boltzmann method (LBM). Compared to previous research, this work overcomes the limitations inherent in traditional studies using natural cores for complex resistivity dispersion analysis. It clearly elucidates the intrinsic relationships between pore structure, fluid properties, and the complex resistivity dispersion in rocks. This research provides valuable references for the interpretation of electromagnetic detection in complex lithology marine reservoirs.

## Methods and principles

**Fig. 1:**
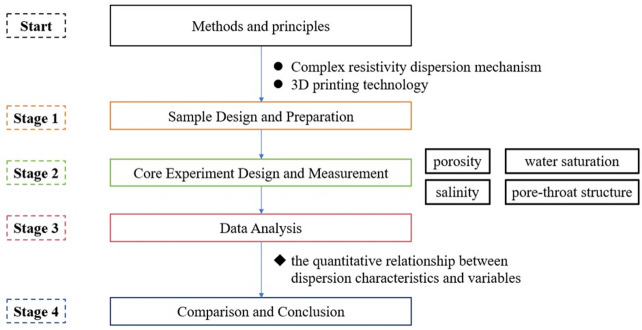
Workflow of this study.

Figure 1 shows the workflow of this study. It begins by introducing the methodological principles involved, including the mechanisms of complex resistivity dispersion and 3D printing technology. Subsequently, the printed core is designed and fabricated, followed by experimental design and measurement on the core. Finally, experimental data are analyzed, and conclusions are discussed and drawn.

### Complex resistivity dispersion mechanism

**Fig. 2:**
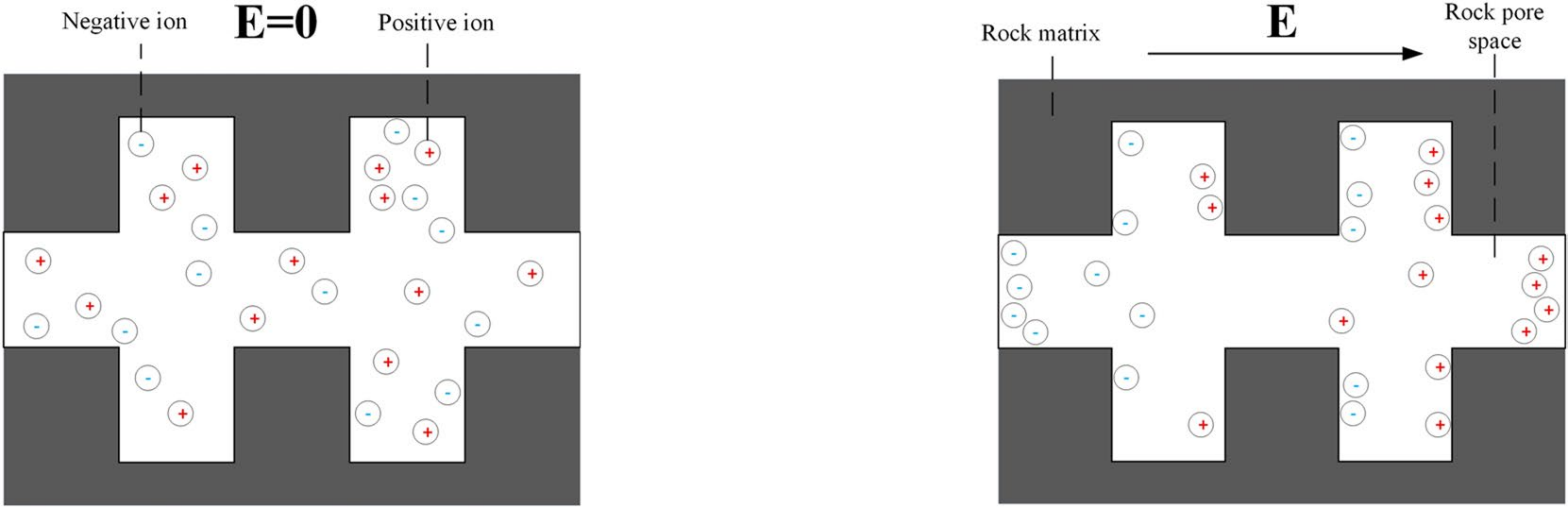
Process of interfacial polarization in rock pores: (**a**) Ion distribution pattern in rock pores without external force; (**b**) Ion distribution pattern in rock pores under the action of electric field force.

Under the action of an external alternating electric field, the constituent particles of matter (such as molecules, ions, and electrons) will be affected by the external electric field and produce electric polarization effects in rock pores The electric polarization effects in porous media mainly include electrode polarization, interfacial polarization, molecular polarization, and electron polarization^39,40^. In the study of complex resistivity dispersion, interfacial polarization plays a major role, and the frequency range of interfacial polarization is 10^3^–10^8^ Hz. The interfacial polarization process is shown in Fig. 2. In the absence of an applied electric field, positive and negative ions are dispersed throughout the pores and generally uncharged. When an external electric field is applied, positive ions will accumulate on the pore walls in the direction of the electric field, while negative ions will accumulate on the pore walls in the inverse direction of the electric field, forming an induced inverse electric field relative to the applied electric field.

In order to further investigate the relationship between the characteristics of complex resistivity dispersion and rock physical properties, core samples with well-defined pore structures need to be fabricated by 3D printing.

### 3D printing technology

**Fig. 3:**
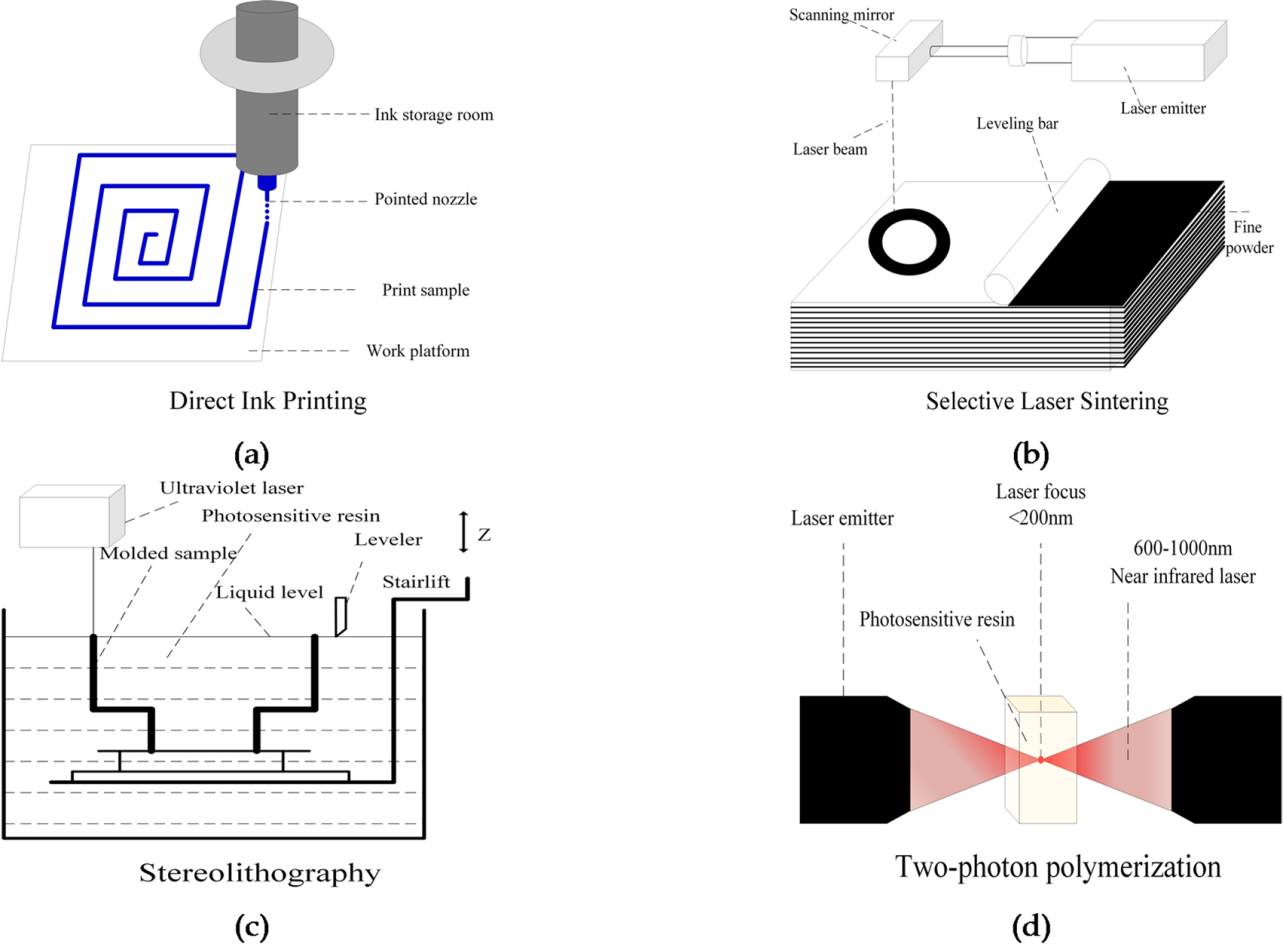
Schematic of 3D printing methods: (**a**) direct ink printing; (**b**) selective laser sintering; (**c**) stereolithography; (**d**) two-photon polymerization.

3D printing technology encompasses a variety of forms and methods. Commonly used 3D printing methods include direct ink writing (DIW), selective laser sintering (SLS), stereolithography (SLA), and two-photon polymerization (TPP)^41–44^. The operating principles of these methods are shown in Fig. 3. DIW squeezes the high-viscosity ink continuously and slowly out from the nozzle through mechanical pressure and creates a pre-set digital model on the printing table. This method features high molding speed and low material cost, but processes such as curing and sintering affect the printing accuracy. SLS produces physical objects by sintering nylon, metal and other powder materials with high-power laser beams. It can use many types of printing materials, but it has disadvantages such as high printing cost and poor surface quality of formed objects. SLA, which is based on photo-curing of liquid photosensitive resin, scans a specified part of liquid photosensitive resin with an ultraviolet laser beam. The area of liquid photosensitive resin scanned by the laser beam will be cured into a thin layer of a specified thickness. The process is repeated until stack printing is completed layer by layer from top to bottom. This method has advantages such as high printing accuracy and high material utilization rate, but it has stringent requirements for the printing environment. Compared with the traditional photo-curing technology, TPP users a near-infrared light source with lower photon energy of 600–1000 nm, and no curing reaction occurs when the single light source is in contact with the photosensitive resin. When the two laser beams are focused at one point, two-photon absorption occurs at the focal point, resulting in polymerization/curing, and the diameter of the focal point is usually less than 200 nm. Therefore, TPP can achieve ultra-high precision 3D printing at the nanoscale, but its printing time is usually ten times that of traditional photo-curing and the dimensions of printed objects are smaller.

Generally, as the printing accuracy improves, the samples that can be manufactured on the printing table will become smaller, and the printing time will be longer. Table 1 shows the values of printing accuracy and printing area of the 3D printing methods described above. Although TPP can reach printing accuracy of 50 nm, the maximum sample molding area is only 2.2 mm×2.2 mm, which is difficult to meet the conditions for the printing of 3D digital core models. Other 3D printing methods such as DIW and fused deposition modeling (FDM) have large printing areas and can meet the dimensional requirements of core sample printing, but their printing accuracy is relatively low, making it difficult for them to accurately print the internal pore structure of core samples, and the accuracy and surface quality of printed cores will be reduced after curing and sintering. In summary, SLA has high printing accuracy, and its maximum printing area can reach 1 m×1 m, which can meet the dimensional requirements of 3D core printing. In addition, for photo-curing technology, photosensitive resin is commonly used as the printing material, the printed core samples have good surface quality, and the model design is characterized by a high degree of freedom. Therefore, SLA was selected as the method for printing 3D cores from digital models in this study.

**Table 1:** Printing accuracy and printing area of 3D printing methods.

3D printing method	Printing accuracy	Printing area
DIW	100 μm–10 cm	30 mm × 30mm–10 m × 10 m
SLS	100 μm–200 μm	5 cm × 5 cm–0.5m × 0.5m
SLA	50 μm–100 μm	1 cm × 1 cm–1 m × 1 m
TPP	50 nm–200 nm	10 μm × 10 μm–2.2 mm × 2. 2mm

## Experiment

Allowing for personalized design and being digital in nature, 3D printing provides a convenient approach to investigate the factors affecting the characteristics of complex resistivity dispersion, and each factor can be controlled as a single variable to study its relationship with complex resistivity dispersion.

### Experiment system

**Fig. 4:**
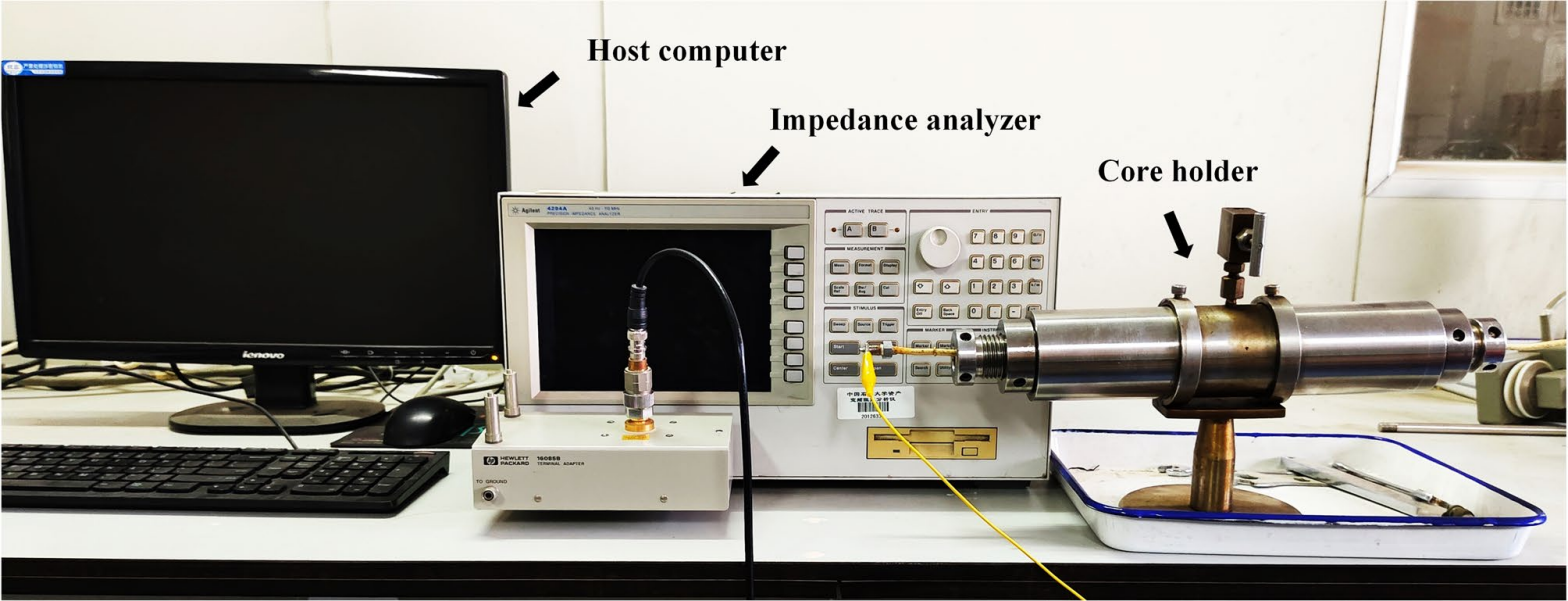
Experimental measurement system.

The experimental measurement system consists of a host computer, an impedance analyzer, and a core holder, as shown in Fig. 4. The host computer is connected to the impedance analyzer and is responsible for setting the parameters of the impedance analyzer and controlling the measurement process. The Agilent 4294 A impedance analyzer is used for impedance measurement and analysis within the frequency range of 40 Hz–110 MHz, with 401 frequency points arranged in a logarithmic distribution. The core holder is provided with two electrodes at the left and right ends, which are connected with the measuring fixture to measure the core impedance.

### Experiment design

Based on the mechanism of complex resistivity dispersion in rocks, experiments were conducted to investigate the effects of pore structure and pore fluid properties, and the effects of porosity, water saturation, salinity, and pore-throat structure on electrical dispersion in printed cores were experimentally measured.

During the selection of salinity levels, four experimental measurement points (1, 5, 10, and 20 kppm, where kppm denotes kilo parts per million, equivalent to 1,000 mg/L) were established. This range was chosen for two primary reasons: (1) the core objective of this study regarding salinity is to investigate the interfacial polarization mechanisms within 3D-printed core samples, and employing a lower salinity range facilitates clearer identification and quantification of electromagnetic responses dominated by solid-liquid interfacial phenomena^45,46^; and (2) concerns regarding the potential for swelling and softening of the 3D-printed material under prolonged exposure to high-salinity brine solutions were a mitigating factor.

For the accurate control of variables, the 3D printed core for each experiment needs to be processed by washing, drying, and saturation of the required brine solution. The specific procedure is detailed below.

Step 1: In the water bath, the core is washed with deionized water by heating and stirring, and the conductivity of the solution in the pot is measured every 12 h. When the conductivity drops to 10 mS/m, the salt washing process can be considered complete.

Step 2: Place the desalted core in an oven at 50 °C and measure the weight of the core every 4 h until the difference between two adjacent measurements is less than 0.01 g.

Step 3: Select four salinity points at 1 kppm, 5 kppm, 10 kppm and 20 kppm, and prepare the NaCl solution required for the experiment.

Step 4: Vacuum the core and soak it in the prepared NaCl solution at 15 Mpa for 72 h until it becomes saturated.

Step 5: Weigh the saturated core, place it in the core holder, and carry out experimental measurement while controlling a single variable each time.

Step 6: Process and analyze the measured spectral data of the core.

During the fifth step, involving saturation variation experiments, the gravimetric method was employed to achieve distinct water saturation states within the core samples. Initially, the mass of the fully saturated core was recorded. Subsequently, pressurized gas was applied to the designated pore-access end of the core to displace the pore water via gas-driven displacement. The mass of the core following this water expulsion phase was then measured. The reduction in mass, corresponding to the mass of expelled water, was utilized to calculate and thus control the target water saturation level.

### Core model design and production

**Fig. 5:**
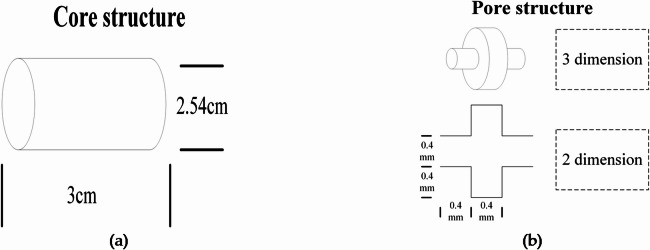
Structure of the core and pore: (**a**) Core structure; (**b**) Pore structure.

First, the overall dimensions and basic unit structure of the designed core were established. As shown in Fig. 5, the overall structure of the core is a cylinder with a diameter of 2.54 cm (1 inch) and a length of 3 cm. The unit structure of pore throats is represented by a capillary bundle model, in which the columnar part of the large hole is the pore and the columnar part of the small hole is the throat. A channel through the whole core is formed through the stacking of pore throats.

According to Fig. 5, several digital core models were designed and established from the perspectives of pore scale and pore structure. The model parameters are summarized in Table 2. The porosity of a digital core is controlled by changing the total number of pore-throat channels on the pore scale. Three digital core models with the same pore throat structure, porosities of 5%, 15% and 25%, and 40, 120 and 200 pore-throat channels were established. The dimensions of pores and throats in rocks are represented by diameter and length. Therefore, 3D digital cores were built based on two diameters, namely, the pore-to-throat length ratio and pore-to-throat diameter ratio. Specifically, three digital core models with pore-to-throat length ratios of 4:1, 2:1 and 1:1 were designed, for which the pore diameter is 1.2 mm, the throat diameter is 0.4 mm, the number of pore-throat channels is 64, and the other parameters are consistent. In addition, four core models with pore-to-throat diameter ratios of 7.5:1, 5:1, 3.75:1 and 3:1 were designed, for which the pore length and throat length are both 0.4 mm, the number of channels is 16, and the other parameters are consistent.

**Table 2:** Core model parameters.

Category	No	Cell pore length/mm	Cell pore diameter/mm	Cell throat length/mm	Cell throat diameter/mm	Number of channels
Variable rock porosity	1	0.4	1.2	0.4	0.4	40
	2	0.4	1.2	0.4	0.4	120
	3	0.4	1.2	0.4	0.4	200
Variable pore throat length	4	0.4	1.2	0.1	0.4	64
	5	0.4	1.2	0.2	0.4	64
	6	0.4	1.2	0.4	0.4	64
Variable pore throat diameter	7	0.4	3.0	0.4	0.4	16
	8	0.4	3.0	0.4	0.6	16
	9	0.4	3.0	0.4	0.8	16
	10	0.4	3.0	0.4	1.0	16

Based on the data in Tables 2 and 10 3D digital core models were built using Auto CAD and printed into samples by SLA using a Uniz NBEED photo-curing 3D printer with a printing accuracy of ± 10 μm and a maximum printing volume of 13.4 × 7.8 × 18 cm. The printing material used is SG photosensitive resin, which is electrically non-conductive and has numerous advantages such as high strength, high precision, and easy cleaning. The whole process consists of two parts: the production of the printed core and the post-processing.

1. Production of printed cores: Select LCD light-curing 3D printer, according to the established digital core model with UNIZ Dental software for slicing processing to generate slice files, and then printed.
2. Post-processing process: The post-processing process is divided into two parts: firstly, the printed core is immersed in alcohol for ultrasonic cleaning for 1–2 min, and then the liquid in the pores is blown dry with compressed air. Subsequently, the core is cured using a post-curing box U Cure, curing conditions wavelength 395 nm laser irradiation lasts for one minute to ensure complete cross-linking of the material to enhance the overall mechanical properties.

**Fig. 6:**
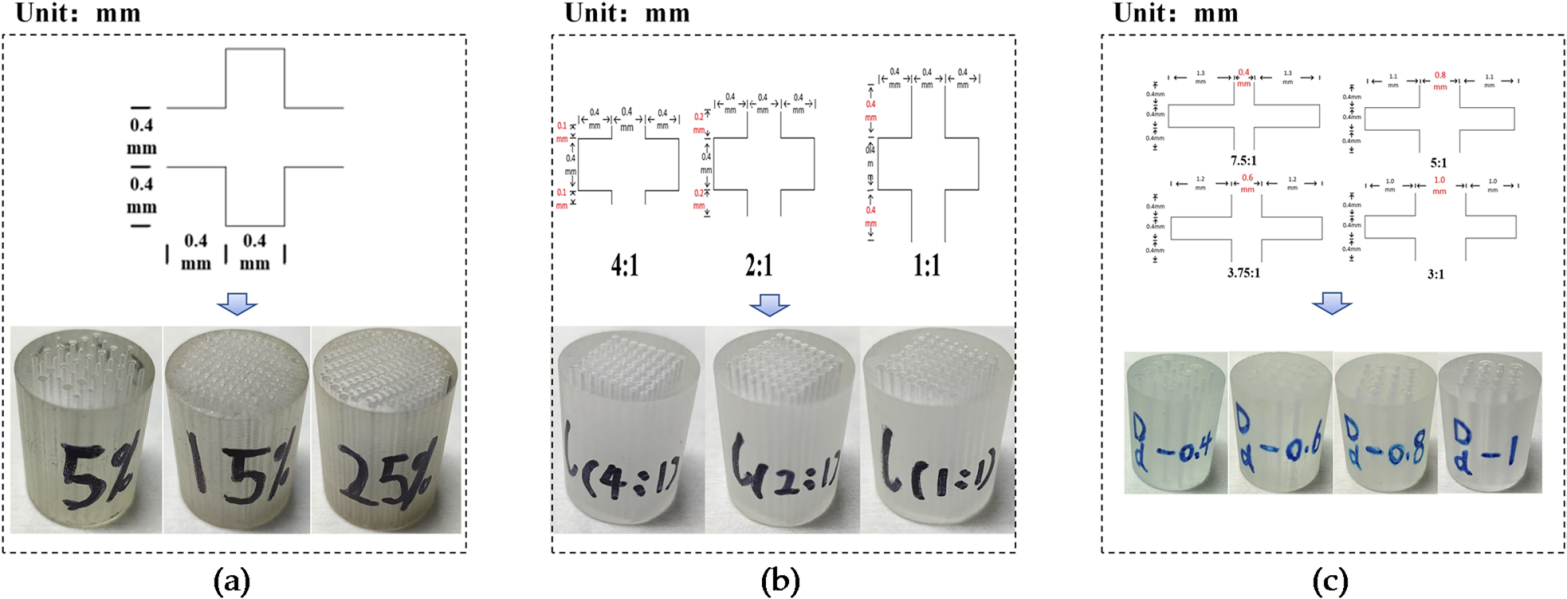
3D printed cores with variable pore-throat parameters: (**a**) Porosity; (**b**) Throat length; (**c**) Throat diameter.

Following the printing process described above, the established digital core model was printed into a physical sample, as shown in Fig. 6.

Three printed samples with the same pore-throat structure and porosities of 5%, 15% and 25% were selected and used to verify the performance of 3D printing methods. Their actual dimensions and porosities were measured, their diameters and lengths were determined by averaging three measurements made with a vernier caliper, and their porosities were measured by the saturated liquid method. The measurement results are shown in Table 3. It can be seen that the diameters of the three core samples are about 2.54 cm, their length are about 3 cm, and their similarity with the theoretical model can reach 99%. In addition, the actual porosities of these core samples are smaller than the theoretical values, and the errors between the actual and theoretical porosity values can be maintained at about 20%. It the errors are converted to differences in pore-throat structure parameters of the samples, such differences will be smaller, and the changes therein at the pore-throat scale will be less than 15%. Therefore, it can be concluded that the 3D printed cores are within the expected tolerances and can be used for electro spectral petrophysical experiments.

**Table 3:** Parameters of 3D printed cores.

No	Calibre (cm)	Length (cm)	Theoretical porosity (%)	Actual porosity (%)	Porosity similarity ratio (%)
1	2.523	3.036	5	4.06	81.20
2	2.524	3.015	15	12.31	82.07
3	2.515	3.001	25	18.92	75.68

## Results

**Fig. 7:**
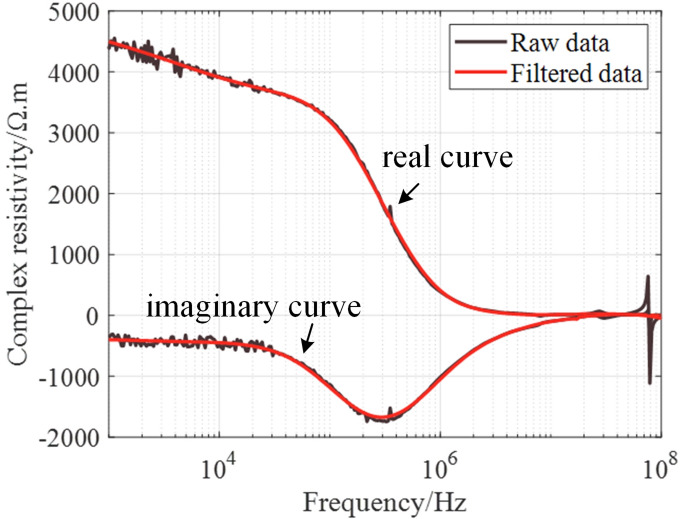
Comparison between the original and filtered experimental data.

When the salinity of the brine in the pores of the core is low during experimental measurement, the experimental data will contain a large amount of noise. In this case, the experimental data needs to be denoised. The denoising method adopted for this study is Savitzky-Golay filtering, which is a filtering method based on local polynomial least squares fitting^47^. It allows for the selection of different window widths at any location and can ensure that the shape and width of the signal remain unchanged during noise elimination, which is very important for spectral analysis. Figure 7 shows the comparison between the original experimental data (raw data) and the data processed by Savitzky-Golay filtering (filtered data). In Fig. 7, the black and red curves represent the original data and the filtered data, respectively. It can be seen that good denoising effects have been achieved, and the characteristic parts of spectral curves have been retained.

### Comparison with actual rock samples

**Fig. 8:**
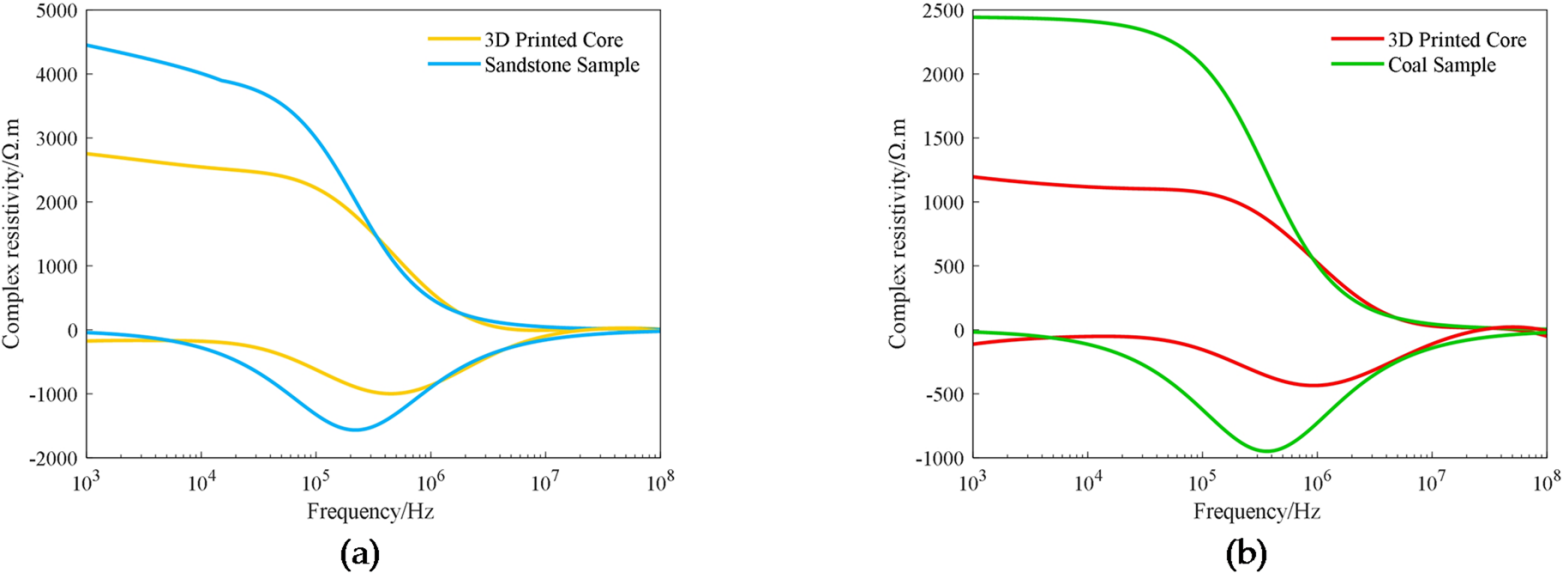
Complex resistivity dispersion comparison between printed cores and actual cores: (**a**) Comparison with sandstone; (**b**) Comparison with coal.

Firstly, we took two 3D printed cores with the same pore structure and porosity of 4.06% and 12.31%, respectively, and compared the differences in the complex resistivity dispersion between them and the artificial sandstone and natural coal samples (as shown in Fig. 8), in which the artificial sandstone has a porosity of 5% and the coal sample has a porosity of 12%. Figure 8 shows that the complex resistivity dispersion law of 3D printed cores is the same as that of the actual cores, in which the real part of the complex resistivity decreases with increasing frequency, and there is an obvious valley frequency in both the imaginary part. The valley frequency is defined as the frequency corresponding to the minimum value in the imaginary part of the complex resistivity spectrum, which can be determined from experimental data.

When analyzing the real part of the complex resistivity, the value at 1 kHz is typically used as the rock’s resistivity, as the influence of electrode polarization generally does not extend beyond this frequency. Analysis of the resistivity values at 1 kHz revealed that the actual rock samples exhibited significantly higher resistivity than the 3D-printed cores. Specifically, whereas the porosity difference between the sandstone and the printed core was 18.8%, their resistivity difference reached 43.3%. Similarly, the porosity difference between the coal sample and the printed core was 2.58%, yet their resistivity difference amounted to 51.12%.

### Effect of porosity

**Fig. 9:**
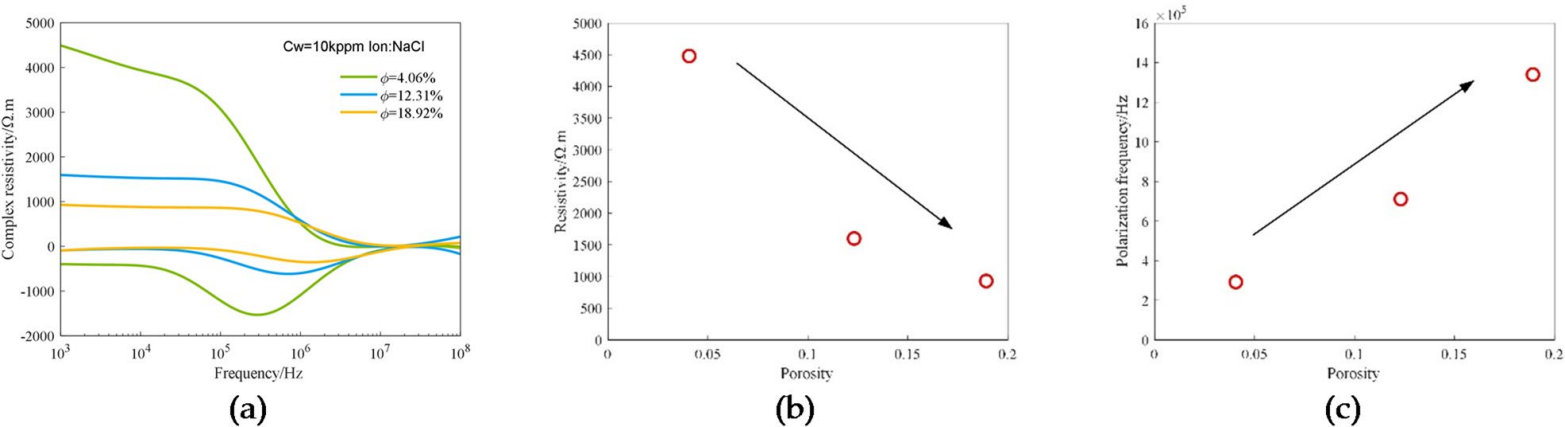
Experimental data obtained under varying porosity conditions: (**a**) The complex resistivity curves corresponding to different porosities; (**b**) The relationship between resistivity (1 kHz) and porosity; (**c**) The relationship between polarization frequency and porosity.

Porosity is one of the most important petrophysical parameters of reservoirs. Three 3D printed cores having the same pore-throat structure and porosities of 4.06%, 12.31% and 18.92% and saturated with 10 kppm NaCl solution were used for experiments, and the spectral data of these samples was obtained through experimental measurement. Figure 9 shows the experimental data obtained under varying porosity conditions. Figure 9(a) shows the complex resistivity curves corresponding to different porosities. It can be seen that, with the increase in porosity, the value of the real part of complex resistivity decreases continuously, and the valley of the imaginary part moves in the direction of frequency increase.

Figure 9(b) and (c) illustrate the observed trends of resistivity and polarization frequency with increasing porosity, under conditions of fixed pore structure. Based on the limited dataset of three porosity samples, resistivity measurements exhibit a decrease with increasing porosity (Fig. 9(b)), while polarization frequency shows an increase with increasing porosity (Fig. 9(c)). It is critical to emphasize that the very small sample size (*n* = 3) constrains these observations solely to indicating directional trends between these parameters under the specific experimental conditions presented. Statistical fitting and correlation coefficient calculations were deliberately omitted, as the limited data are insufficient to support reliable quantitative inferences. Consequently, these results should be interpreted as preliminary observations of potential trends, rather than as established quantitative relationships.

### Effect of water saturation

**Fig. 10:**
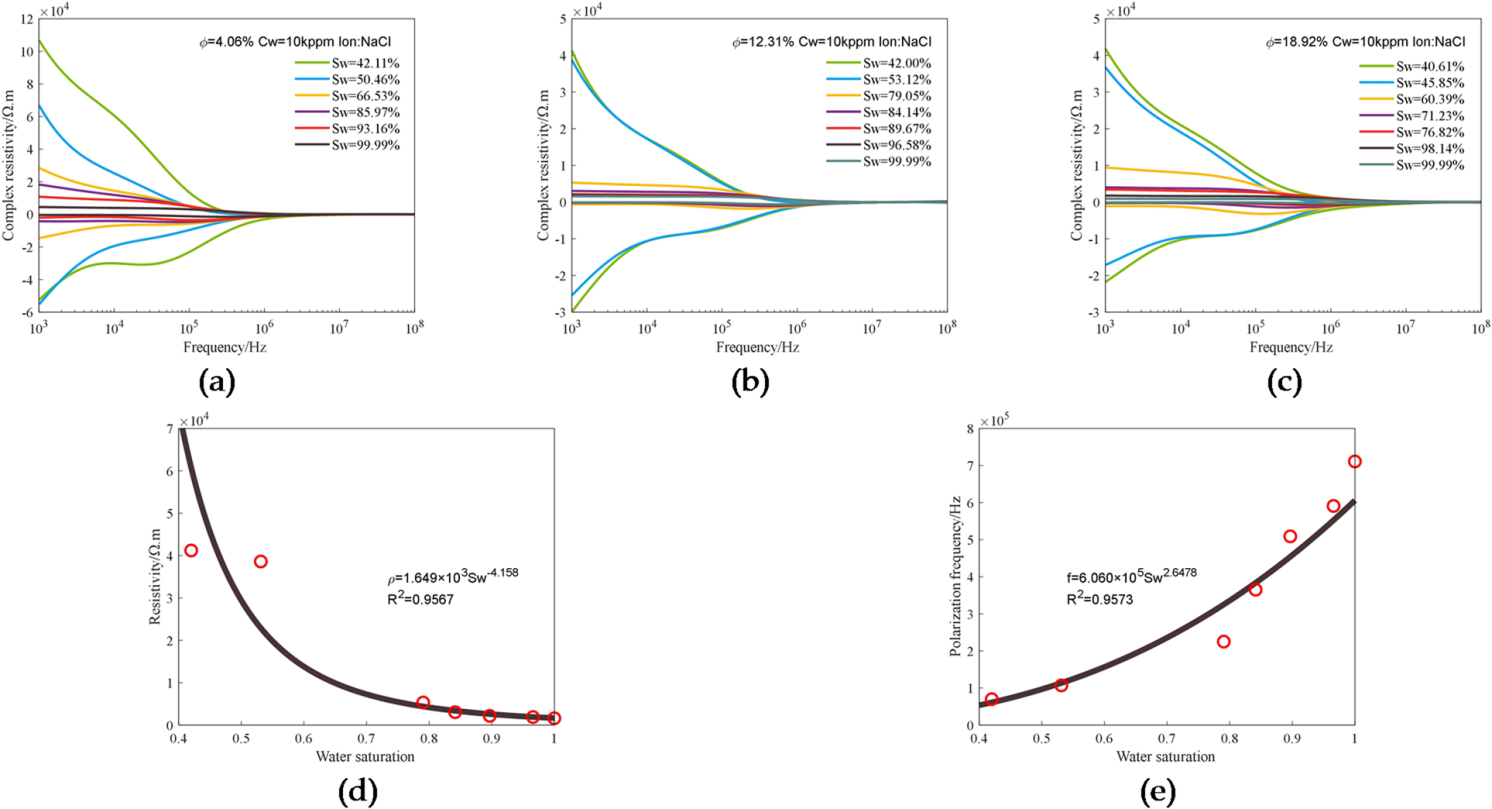
Experimental data obtained under varying water saturation conditions: (**a**) The complex resistivity curves corresponding to different water saturations in a core with 4.06% porosity; (**b**) The complex resistivity curves corresponding to different water saturations in a core with 12.31% porosity; (**c**) The complex resistivity curves corresponding to different water saturations in a core with 18.92% porosity; (**d**) The relationship between resistivity (1 kHz) and water saturation in a core with 12.31% porosity; (**e**) The relationship between polarization frequency and water saturation in a core with 12.31% porosity.

Water saturation is a key factor in geological evaluation that directly affects the core, oil content and production value of the reservoir considered, and it is also an important parameter for evaluating the petrophysical properties of reservoirs. Three 3D printed cores having the same pore structure and different porosities of 4.06%, 12.31 and 18.92% and saturated with 10 kppm NaCl solution were experimentally measured under varying water saturation conditions. The water saturation of the cores was changed by gas displacement, and the electro-spectral curves were measured under varying water saturation conditions^48^. Figure 10 shows the experimental data obtained under different water saturation conditions. Figure 10a–c show changes in complex resistivity of the three cores with water saturation. It can be seen that, for all of the three cores, with the increase in water saturation, the value of the real part of complex resistivity decreases, and the frequency valley of the imaginary part moves in the direction of frequency increase.

The relationship between resistivity and water saturation and the relationship between polarization frequency and water saturation determined from the experimental data corresponding to 12.31% porosity are shown in Fig. 10(d) and (f). It can be seen that there is a good exponential relationship between water saturation and resistivity, in which the latter decreases exponentially with the former, the absolute value of the power is 4.158, and the fitting degree is 92.3%; there is a relatively good exponential relationship between water saturation and polarization frequency, in which the latter increases exponentially with the former, the absolute value of the power is 2.648, and the fitting degree is 95.7%.

### Effect of salinity

**Fig. 11:**
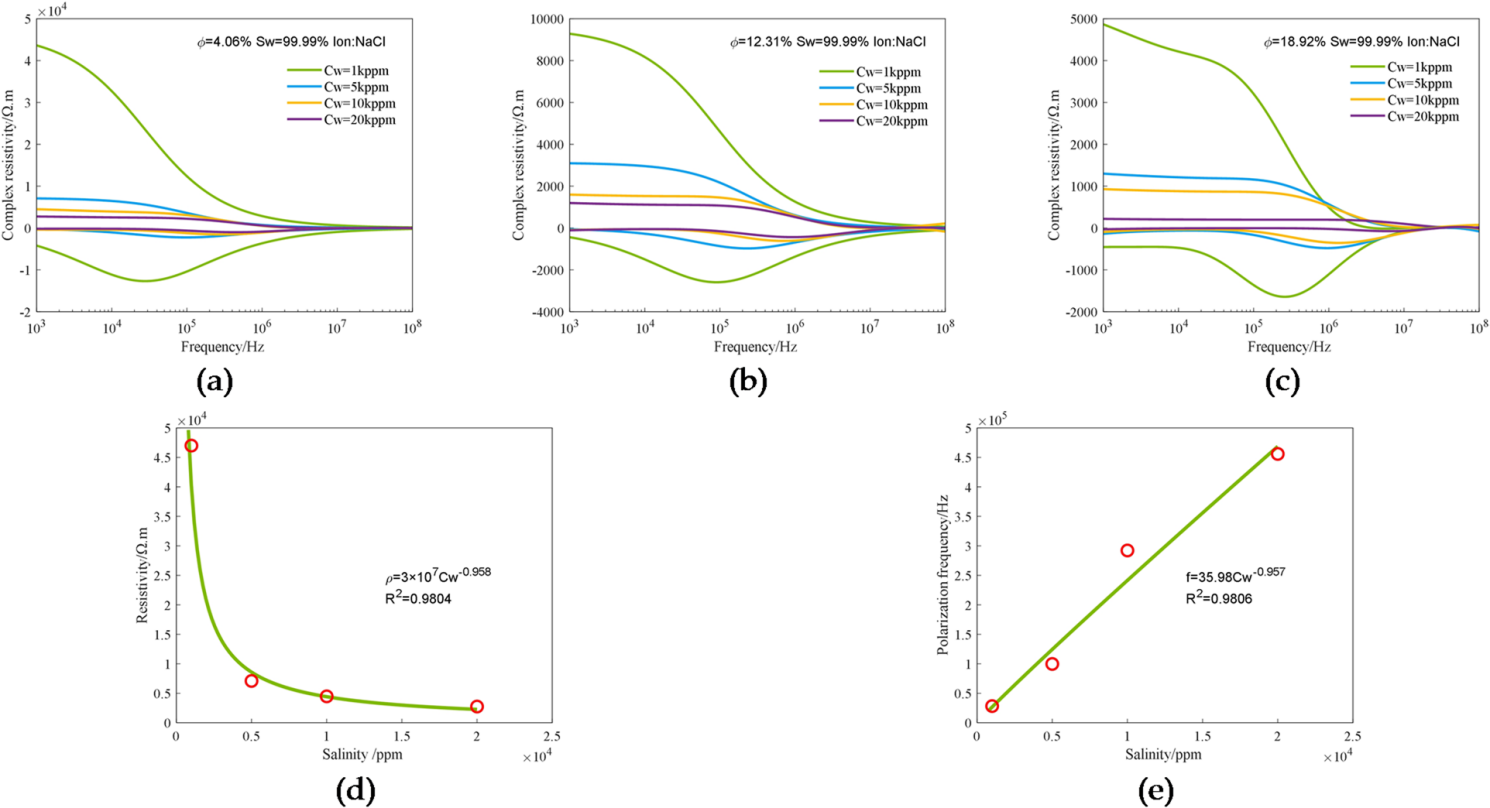
Experimental data obtained under varying salinity conditions: (**a**) The complex resistivity curves corresponding to different salinities in a core with 4.06% porosity; (**b**) The complex resistivity curves corresponding to different salinities in a core with 12.31% porosity; (**c**) The complex resistivity curves corresponding to different salinities in a core with 18.92% porosity; (**d**) The relationship between resistivity (1 kHz) and salinity in a core with 4.06% porosity; (**e**) The relationship between polarization frequency and salinity in a core with 4.06% porosity.

The change in formation water salinity will increase the complexity and difficulty of reservoir evaluation. Therefore, the experimental study of the effect of salinity on electrical dispersion in rocks is very important for accurate reservoir evaluation. Three 3D printed cores with the same pore-throat structure and different porosities of 4.06%, 12.31% and 18.92% were experimentally measured under varying salinity conditions, and specifically, the electro-spectral curves of cores saturated with 1 kppm, 5 kppm, 10 kppm, and 20 kppm NaCl solutions were measured. Figure 11 shows the analysis of the experimental data obtained under varying salinity conditions, in which Fig. 11a–c show the changes in complex resistivity of the three printed cores with salinity. It can be seen that, for all the three cores, with the increase in salinity, the value of the real part of complex resistivity decreases, and the frequency valley of the imaginary part moves in the direction of frequency increase.

The relationship between resistivity and salinity and the relationship between polarization frequency and salinity determined from the experimental data corresponding to 4.06% porosity are shown in Fig. 11(d) and (e). It can be seen that there is a relatively good exponential relationship between salinity and resistivity, in which the latter decreases exponentially with the former, the absolute value of the power is 0.958, and the fitting degree is 98.04%; there is a good exponential relationship between salinity and polarization frequency, in which the latter increases exponentially with the former, the absolute value of the power is 0.957, and the fitting degree is 98.06%.

### Effect of pore-throat structure

**Fig. 12:**
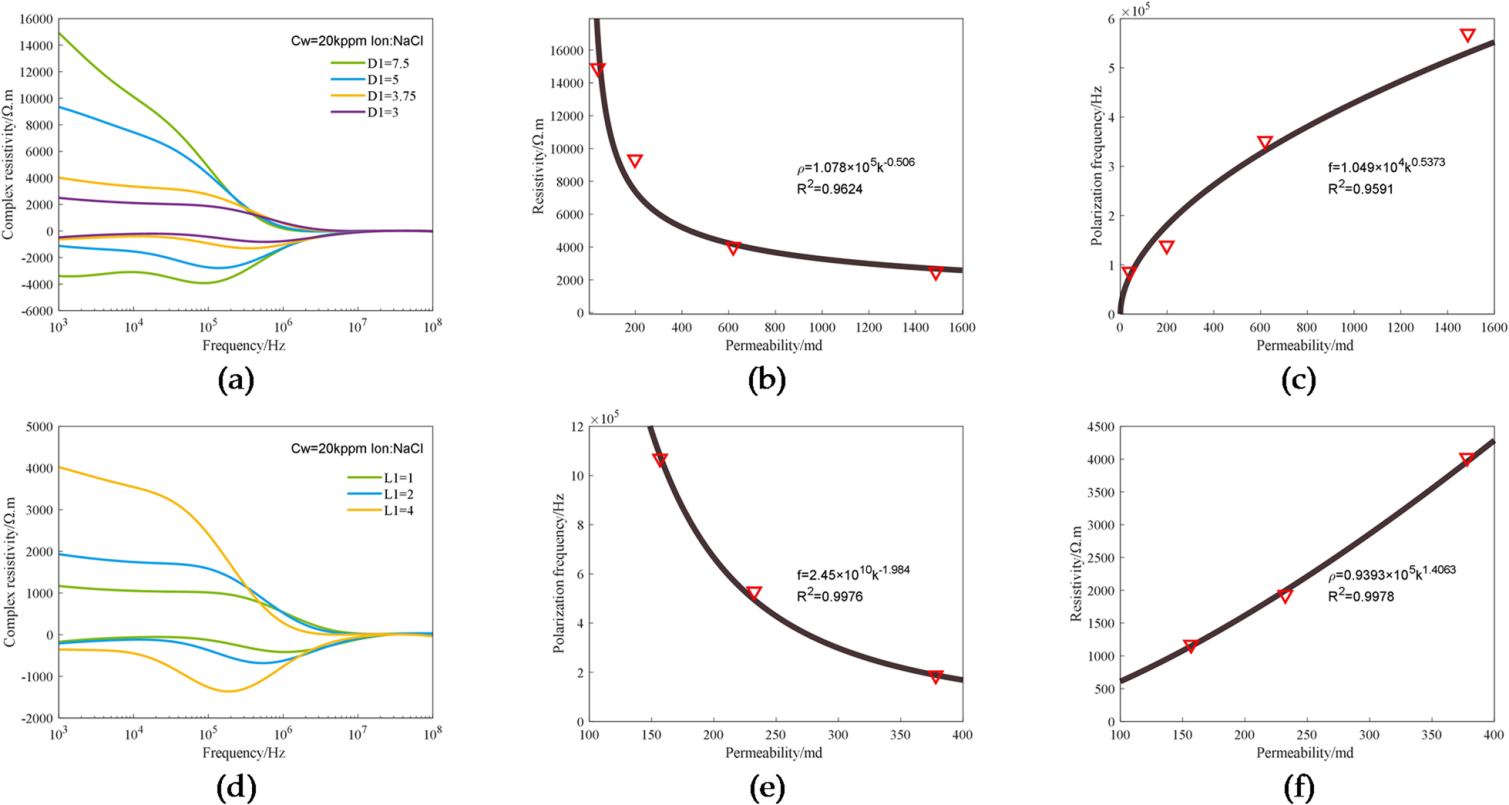
Experimental data for 3D printed cores with different pore-throat structures: (**a**) The complex resistivity curves corresponding to pore-to-throat diameter ratio; (**b**) The relationship between resistivity (1 kHz) and permeability in different pore-to-throat diameter ratios; (**c**) The relationship between polarization frequency and permeability in different pore-to-throat diameter ratios; (**d**) The complex resistivity curves corresponding to pore-to-throat length ratio; (**e**) The relationship between resistivity (1 kHz) and permeability in different pore-to-throat length ratios; (**f**) The relationship between polarization frequency and permeability in different pore-to-throat length ratios.

The electrical dispersion properties of rocks, especially interfacial polarization, are closely related to the pore structure of rocks. In particular, the interfacial polarization relaxation time varies with pore structure. In order to investigate the relationship between pore structure and electrical dispersion, experiments were conducted on seven 3D printed cores designed based on the pore-to-throat diameter ratio and pore-to-throat length ratio and saturated with 20 kppm NaCl solution, and their electric-spectral curves were measured. We define the ratio of the length of the pore to the throat as L1; the ratio of the diameter of the pore to the throat as D1. Figure 12 shows the analysis of experimental data obtained under conditions of different pore-throat structures. Figure 12(a) shows the relationship between the pore-to-throat diameter ratio and complex resistivity. It can be seen that, as the throat diameter increases, the value of the real part of complex resistivity decreases, and the valley of the imaginary part moves in the direction of frequency increase. From the core design, it can be known there are no significant difference in theoretical porosity between the four printed cores with different pore-throat diameters, and due to the change in throat diameter, there are great differences in permeability between the cores. The relationship between the designed capillary-bundle core model and permeability can be expressed as^49^:

1 $$k=\frac{{{n_0}\pi }}{{128}}{D_{\mathrm{s}}}^{4}\frac{{1+y}}{{1+y/{x^4}}}$$

where ${n_0}$ is the number of capillaries per unit cross-sectional area, *y* is the pore-to-throat diameter ratio, *x* is the pore-to-throat length ratio, ${D_{\mathrm{s}}}$ is the throat diameter, and *k* represents permeability.

The permeability values of cores with different pore-throat diameters calculated using the equation above are 39.53 mD, 198.43 mD, 619.04 mD, and 1486.53 mD, respectively.

The relationship between resistivity and permeability and the relationship between polarization frequency and permeability determined based on the calculated permeability values are shown in Fig. 12(b) and (c). There is a good exponential relationship between permeability and resistivity, in which the latter decreases exponentially with the former, the absolute value of the power is 0.506, and the fitting degree is 96.24%; there is a good exponential relationship between permeability and polarization frequency, in which the latter increases exponentially with the former, the absolute value of the power is 0.5373, and the fitting degree is 95.91%.

Figure 12(d) shows the relationship between the pore-to-throat length ratio and complex resistivity. It can be seen that, as the throat length increases, the value of the real part of complex resistivity decreases, while the polarization frequency valley of the imaginary part moves in the direction of frequency increase. The permeability values of cores with pore-to-throat length ratios of 1:1, 2:1 and 4:1 are 156.78 mD, 232.34 mD, and 378.14 mD, respectively.

Figure 12(e) and (f) shows the relationship between resistivity and permeability and the relationship between polarization frequency and permeability for 3D printed cores with different pore-throat lengths. As can be seen from the figure, for these cores, there is a good exponential relationship between resistivity and permeability, in which the former increases exponentially with the latter, the absolute value of the power is 1.401, and the fitting degree is 99.8%; there is a good exponential relationship between polarization frequency (imaginary part) and permeability, in which the polarization frequency decreases exponentially with the increase in permeability, the absolute value of the power is 1.984, and the fitting degree is 99.7%.

### Simulation-based verification

The experimentally identified rules of electrical dispersion in 3D printed cores with different pore-throat structures show that, although the characteristics of electrical dispersion in rocks have regular relationships with macroscopic parameters such as porosity and permeability, the real influencing factors should be related to the microscopic behavior of ions in rock pores under the action of an external electric field. The Lattice Boltzmann method (LBM), as a mesoscopic statistical method between macroscopic continuous methods and microscopic dynamic methods, explores the distribution state of particles under external forces from a mesoscopic perspective to reflect the macroscopic properties of the problem, and it has been widely studied and used in a large number of fields, such as multiphase flow, porous media, and heat transfer^50,51^. Shi et al. verified the applicability of LBM to the study of electrical dispersion in rocks using Archie’s formula and Darcy’s law. In order to explain the phenomenon of contradictory rules in pore structure-related experiments, simulation-based verification was performed from a mesoscopic perspective using LBM.

According to the differential form of Maxwell’s equations and its constitutive equation, the evolution equation for the current density can be derived.

2 $$\frac{{\partial \rho }}{{\partial t}}+\nabla \cdot \vec {J}=0$$

where $\rho$ is the charge density, and $\vec {J}$ is the current density. In an ideal conductive fluid, electric charges migrate under the action of an electric field, and the constitutive relation for the current density is as follows^52^:

3 $$\vec {J}=\rho K\vec {E} - D\nabla \rho +\rho \vec {u}$$

where *K* is the ion mobility and *D* is the diffusion coefficient.

The current density consists of three parts: ion migration in the electric field, molecular diffusion, and fluid micro-cluster flow. In the study of electric dispersion in rocks, it can be considered that it is mainly affected by ion migration in the electric field, and the evolution equation for the charge density is as follows:

4 $$\frac{{\partial \rho }}{{\partial t}}+K\nabla \cdot \rho \vec {E}=0$$

The equilibrium distribution function is the key factor for the application of LBM to different problems. As long as a suitable equilibrium distribution function is provided, different physical problems can be solved by LBM. In the study of complex resistivity dispersion, the charge density corresponds to the ion density of the pore fluid(s), and the equilibrium distribution function under the action of an external electric field can be obtained^53^:

5 $$f_{i}^{{eq}}=\rho {w_i}\left[ {1+\frac{{2{{\vec {c}}_i} \cdot K\vec {E} - {{(K\vec {E})}^2}}}{{2c_{s}^{2}}}+\frac{{{{({{\vec {c}}_i} \cdot K\vec {E})}^2}}}{{2c_{s}^{4}}}} \right]$$

Where $f_{i}^{{eq}}$ is the equilibrium distribution function, ${w_i}$ is the weight coefficient, and ${c_s}$ is the lattice sound velocity.

**Fig. 13:**
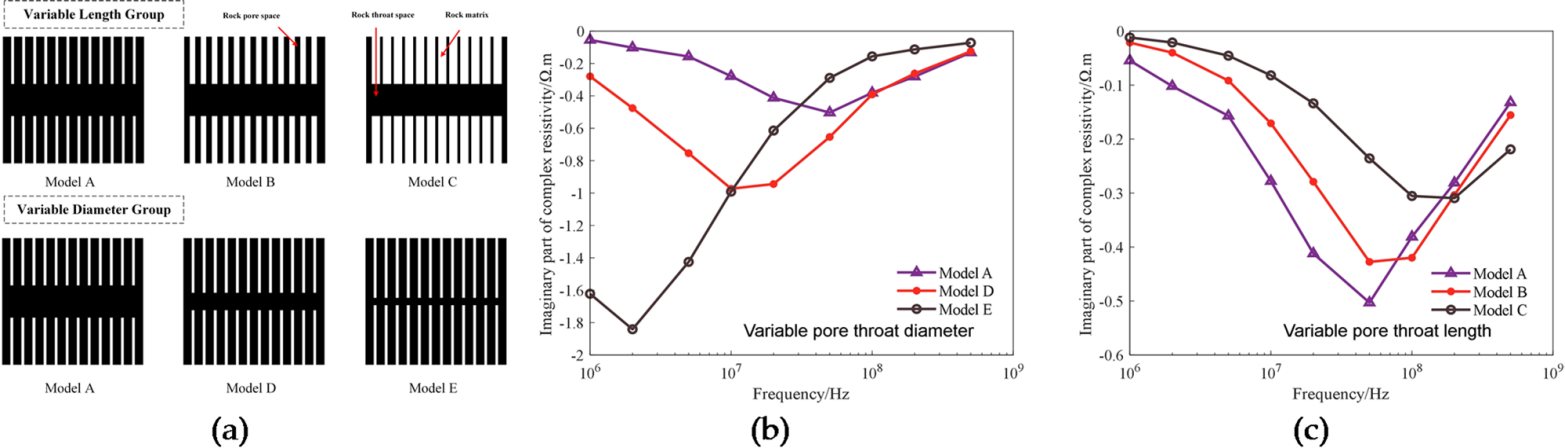
2D lattice models and LBM simulation results: (**a**) lattice models with different throat lengths and diameters ; (**b**) The relationship between imaginary part of complex resistivity and variable pore throat diameter; (**c**) The relationship between imaginary part of complex resistivity and variable pore throat length.

Based on the theory above, five 2D lattice models with different throat lengths and diameters were built by changing the length and width of matrix lattices. Figure 13(a) show the schematic diagrams of these lattice models. The lattice size of these models is 51*51, the black lattices represent pore/pore-throat channels, and the white ones represent the rock matrix. The numerical simulation of these 2D models was performed using LBM at nine frequency points, namely, 500, 200, 100, 50, 20, 10, 5, 2 and 1 MHz. The simulation results are presented in Fig. 13(b) and (c). Figure 13(b) shows the numerical experimental response of variable orifice throat diameter, when the diameter of the throat becomes small, the imaginary part of the complex resistivity moves to the direction of frequency decrease; Fig. 13(c) shows the numerical experimental response of variable orifice throat length, when the length of the throat becomes small, the imaginary part of the complex resistivity moves to the direction of frequency increase. It can be seen that the rule revealed by numerical simulation is consistent with the experimentally identified rule described in the subsection 4.5, which verifies the foregoing explanation about the change in pore-throat structure is correct.

**Fig. 14:**
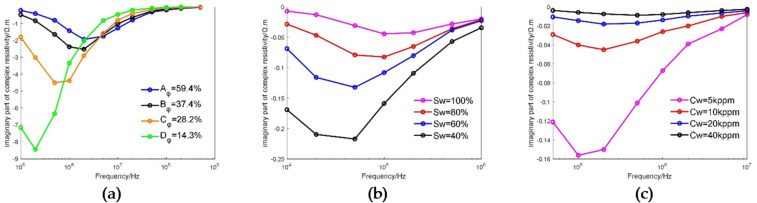
LBM simulation results: (**a**) The relationship between imaginary part of complex resistivity and variable porosity ; (**b**) The relationship between imaginary part of complex resistivity and variable water saturation; (**c**) The relationship between imaginary part of complex resistivity and variable salinity.

Additionally, since the observed trends in porosity, saturation, and salinity align with those reported in previous experimental studies, we conducted a simple validation of our findings regarding porosity, saturation, and salinity. For the porosity simulation, four lattice models with varying porosity levels were constructed. Saturation was controlled by adjusting the proportion of pore-space lattice points available for particle movement according to the target saturation level, while mineralization was simulated by modifying the ion density at each lattice point. The resulting simulation trends, as shown in Fig. 14, corroborate the experimental observations from 3D-printed core samples.

### Sensitivity analysis

Sensitivity analysis is an analytical technique to study the effect of a certain change in the relevant factors on one or a group of key indicators, and it can evaluate the degree of influence of each factor on the compound resistivity of the rock. In this regard, we set a sensitivity coefficient *S*, which is used to evaluate the degree of influence of each relevant factor on the complex resistivity. The sensitivity factor *S* is defined as:

6 $$S=\frac{{\Delta A/A}}{{\Delta F/F}}=\frac{{({A_1} - {A_2})/{A_2}}}{{({F_1} - {F_2})/{F_2}}}$$

In the formula, the larger the absolute value of S, the more sensitive *A* is to *F*; conversely, the less sensitive *A* is to *F*. In this study, *F* is the resistivity and polarization frequency, respectively, because the permeability parameter will change with the change of porosity parameter, and the two are the relationship of mutual influence, so this paper’s sensitivity analysis is only to consider the porosity. *A* is the porosity, the water saturation, and the mineralization parameter, respectively.

**Fig. 15:**
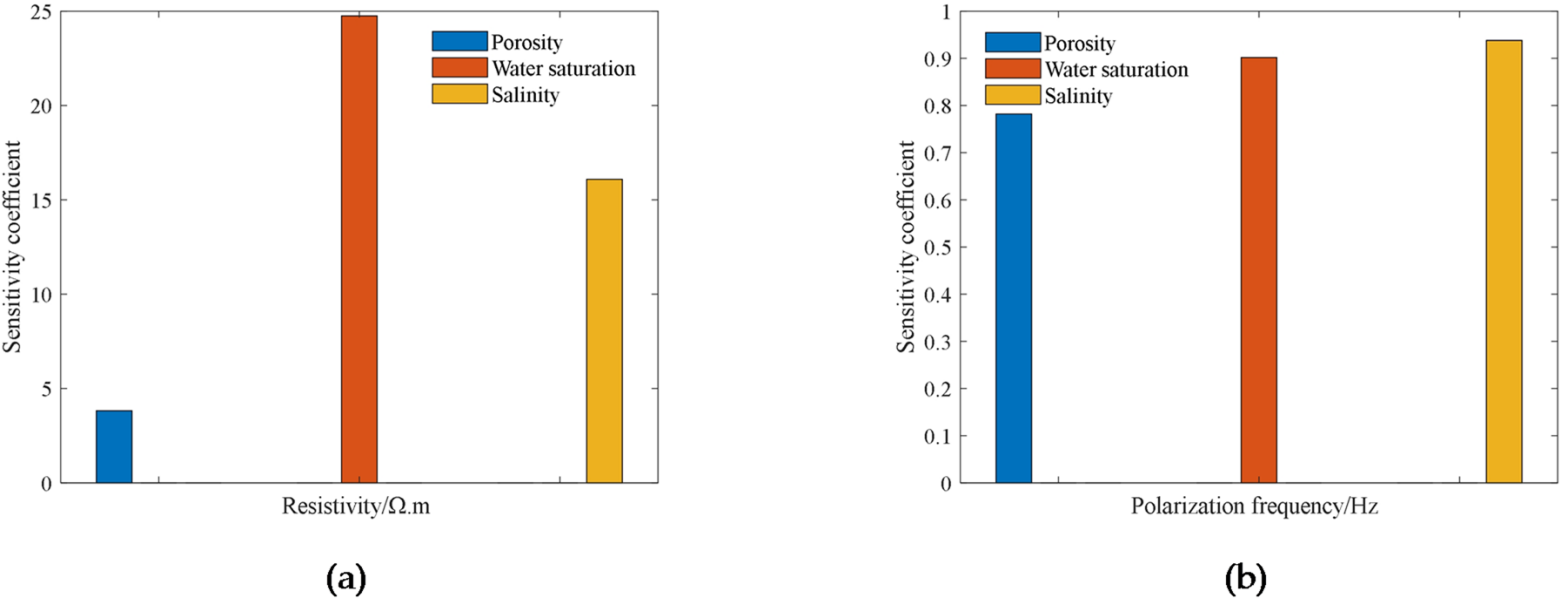
Sensitivity analysis: (**a**) Resistivity versus sensitivity coefficient; (**b**) Polarization frequency versus sensitivity coefficient.

Through the calculation, statistics and analysis of the core data, the combined sensitivity of core resistivity and polarization frequency to each influencing factor was obtained, as shown in Fig. 15(a). From Fig. 15(a), the sensitivity coefficient of rock resistivity to water saturation is the largest and much larger than that of core resistivity to porosity. From Fig. 15(b), the sensitivity coefficient of rock polarization frequency to mineralization is the largest, followed by water saturation, and the smallest is porosity. Therefore, for core resistivity and polarization frequency, the sensitivity of the two to each influencing factor has the following relationship.

7 $$Sw \approx Cw>pro$$

In the equation, $Sw$, $Cw$, and $pro$ represent the sensitivity of the petrophysical electrical parameter to water saturation, water salinity, and porosity, respectively.

## Discussion

Our findings demonstrate significant correlations between the complex resistivity dispersion of 3D-printed cores and variations in porosity, saturation, salinity, and critically, pore structure, the latter validated through numerical modeling. A detailed discussion of the underlying physical mechanisms driving these observed relationships is warranted. The inherent limitations of the experimental methodology are also addressed, laying the groundwork for future research directions.

An increase in core porosity, saturation, and salinity elevates ion concentration within the pore space. Under an applied alternating electric field, this heightened ion concentration reduces the characteristic interfacial polarization relaxation time, consequently shifting the frequency corresponding to the minimum in the imaginary component of the complex resistivity to higher values.

An increase in throat diameter significantly enhances core permeability, facilitating the transport of pore fluids and ions. This process reduces the magnitude of the real part of the complex resistivity and elevates the characteristic polarization frequency of the imaginary part. Under these conditions, permeability exerts a more dominant influence than porosity on the frequency dispersion characteristics of complex resistivity. Conversely, variations in throat length exhibit an opposite trend. While a reduction in throat length similarly increases permeability, it induces reverse changes in the electrical response. The underlying mechanism lies in the fact that shorter throats increase the number of pore-throat units per unit volume. For 3D-printed core models with identical pore space, low-frequency excitation provides sufficient relaxation time for ions to accumulate at the pore-throat interfaces, generating significant interfacial polarization effects. This effect induces strengthened polarization electric fields in opposing directions between adjacent units, thereby increasing the overall resistivity of the core.

The results presented in Fig. 8 reveal significant differences in resistivity between the photosensitive resin cores and the actual rock minerals. However, due to the inherent disparity in resistivity between the resin material and the constituent minerals of natural rock cores, these differences inevitably introduce limitations and potential bias into the measured results. Nevertheless, utilizing 3D-printed photosensitive resin cores with well-defined and regular pore structures demonstrates the capability to reproduce the characteristic complex resistivity dispersion patterns observed in natural rock samples. This indicates the potential of 3D printing technology for elucidating the underlying physical mechanisms of electrical dispersion in rocks, particularly concerning the effects of pore structure.

Li et al. established an exponential relationship between porosity and both resistivity and polarization frequency through complex resistivity measurements on synthetic sandstone samples in 2019^54^. This finding exhibits certain discrepancies with the experimental results presented in Fig. 9. These differences likely arise primarily from variations in pore structure control. For the synthetic cores investigated by Li et al., an increase in porosity necessarily coincided with significant alterations in pore throat size, shape, tortuosity, and connectivity – that is, an increase in pore structure complexity^55,56^. This evolution in pore structure is the key factor responsible for the non-linear variations of resistivity and polarization frequency with porosity. A primary objective of this study was to utilize 3D printing technology to fix the pore structure (geometric morphology) while systematically varying only the porosity (volume fraction). Under these highly controlled, albeit simplified, conditions, we observed a monotonic decrease in resistivity and a monotonic increase in polarization frequency with increasing porosity. This suggests that, when the major confounding factor of concurrent pore structure complexification is significantly minimized, porosity may exert a more direct and relatively monotonic influence on resistivity and polarization frequency.

However, despite our intent to fix the pore structure, an increase in pore volume, even with identical macroscopic structural designs, may introduce unavoidable subtle variations at the microscale due to inherent limitations of the 3D printing process and other uncontrolled factors. Consequently, it should be emphasized that the results of Fig. 9 must be interpreted as demonstrating directional trends: namely, a decrease in resistivity and an increase in polarization frequency with increasing porosity, under the condition that the macroscopic geometry of pore throats was fixed as consistently as possible using 3D printing technology. This observation aligns with the view that increasing pore structure complexity is the primary driver of non-linear responses. Nevertheless, rigorously isolating the effects of porosity from those of pore structure would necessitate a significant expansion of the sample size and the development of more refined printing or characterization techniques to ensure microstructural consistency.

## Conclusion

In this study, artificial cores with precisely controlled pore structures were successfully fabricated using 3D printing technology. We systematically investigated the relationships between the complex resistivity dispersion and key influencing factors—namely porosity, water saturation, salinity, and pore-throat structure. The experimental findings were further validated through lattice Boltzmann method (LBM) simulations. The key findings demonstrate that resistivity and polarization frequency exhibit a monotonic relationship with porosity, but vary exponentially with water saturation and salinity. More importantly, it was discovered that the throat-to-pore diameter ratio and the throat-to-pore length ratio exert opposing influences on complex resistivity dispersion. This highlights that macroscopic parameters such as porosity and permeability are not the sole determinants; the microscopic pore structure plays a critical role. Furthermore, this study established quantitative relationships between pore structure parameters (L1 and D1) and complex resistivity dispersion parameters, thereby clarifying the contribution of pore geometry. These findings provide valuable data to enhance the accuracy of well-log interpretation. For future work, it would be meaningful to integrate these quantitative relationships into more sophisticated petrophysical models and to extend the methodology to a wider variety of rock types.

## Data Availability

The datasets used and/or analysed during the current study available from the corresponding author on reasonable request.
